# Global changes in the pattern of connectivity in developmental prosopagnosia

**DOI:** 10.1093/cercor/bhae435

**Published:** 2024-11-07

**Authors:** Gabriela Epihova, Richard Cook, Timothy J Andrews

**Affiliations:** MRC Cognition and Brain Sciences Unit, University of Cambridge, Cambridge, CB2 7EF, United Kingdom; Department of Psychology, University of York, York, YO10 5DD, United Kingdom; Department of Psychology, University of York, York, YO10 5DD, United Kingdom; School of Psychology, University of Leeds, Leeds, LS2 9JT, United Kingdom; Department of Psychology, University of York, York, YO10 5DD, United Kingdom

**Keywords:** face recognition, developmental prosopagnosia, functional connectivity, structural connectivity

## Abstract

Developmental prosopagnosia is a neurodevelopmental condition characterized by difficulties in recognizing the identity of a person from their face. While current theories of the neural basis of developmental prosopagnosia focus on the face processing network, successful recognition of face identities requires broader integration of neural signals across the whole brain. Here, we asked whether disruptions in global functional and structural connectivity contribute to the face recognition difficulties observed in developmental prosopagnosia. We found that the left temporal pole was less functionally connected to the rest of the brain in developmental prosopagnosia. This was driven by weaker contralateral connections to the middle and inferior temporal gyri, as well as to the medial prefrontal cortex. The pattern of global connectivity in the left temporal pole was also disrupted in developmental prosopagnosia. Critically, these changes in global functional connectivity were only evident when participants viewed faces. Structural connectivity analysis revealed localized reductions in connectivity between the left temporal pole and a number of regions, including the fusiform gyrus, inferior temporal gyrus, and orbitofrontal cortex. Our findings underscore the importance of whole-brain integration in supporting typical face recognition and provide evidence that disruptions in connectivity involving the left temporal pole may underlie the characteristic difficulties of developmental prosopagnosia.

## Introduction

Prosopagnosia refers to the inability to recognize the identity of a person from their face despite intact visual acuity and otherwise typical perceptual abilities. In cases of acquired prosopagnosia, individuals initially develop normal face recognition abilities, but following damage to regions of the occipital and temporal lobes, experience profound difficulties in recognizing people from their faces (Barton 2008). In contrast, a subset of the population experiences significant lifelong difficulties in face recognition without any history of brain injury or trauma (Duchaine and Nakayama 2006a; Susilo and Duchaine 2013). This condition, termed developmental prosopagnosia (DP), is characterized by face-processing difficulties that are thought to emerge early in childhood and persist throughout life, suggesting a neurodevelopmental basis for the disorder.

Several studies have investigated the neural basis underlying face recognition difficulties in individuals with DP, focusing on face-selective regions of the human brain (Duchaine and Nakayama 2006a; Avidan and Behrmann 2014). The occipital face area (OFA), fusiform face area (FFA), and superior temporal sulcus (STS) comprise a core network that is preferentially involved in face perception (Kanwisher et al. 1997; Haxby et al. 2000; Kanwisher 2010). Some studies have found reduced activity in the core face-selective areas when viewing faces in DP (Hadjikhani and De Gelder 2002; Furl et al. 2011; Jiahui et al. 2018); however, others find activity comparable to that of neurotypical individuals (Hasson et al. 2003; Avidan et al. 2005; Rivolta et al. 2014). The core face regions in the posterior temporal lobe are connected to an extended network of regions in the brain that process faces (Haxby et al. 2000; Gobbini and Haxby 2007; Ishai 2008; Davies-Thompson and Andrews 2012). The interaction between the core and extended networks in the temporal lobe is thought to be important for specific aspects of face perception, such as identity (Rotshtein et al. 2005) and expression (Harris et al. 2012).

An alternative explanation for the deficit in face recognition in DP is that it results from a disruption in the connectivity between the core and extended regions of the face network (Fox et al. 2008; Thomas et al. 2009; Behrmann and Plaut 2013; Avidan et al. 2014; Zhao et al. 2018; Sokolowski and Levine 2023). Recently, Levakov et al. (2023) demonstrated that less functional connectivity to the anterior temporal cortex is associated with worse face recognition performance in a neurotypical population. Consistent with this, DPs also show reduced functional connectivity to the anterior temporal cortex (Rosenthal et al. 2017). Noad et al. (2024) reported even more widespread reductions of connectivity in DP involving both the core face network and an extended familiarity network across the whole brain. Evidence supports the idea that this reduction in functional connectivity, to some extent, might be a consequence of a structural alteration in white matter tracts. Two large white matter tracts—the inferior longitudinal fasciculus (ILF) and the inferior fronto-occipital fasciculus (IFOF) connect the occipital to the temporal and frontal cortex, respectively. Alterations in the structure of these long-range tracts (Thomas et al. 2009; Grossi et al. 2014;) as well as local reductions in the vicinity of face-selective regions along the tracts (Gomez et al. 2015; Song et al. 2015) have been reported in DP.

The ability to recognize and then interact appropriately with people that we know requires the integration of visual information with nonvisual episodic, semantic, and affective information (Gobbini and Haxby 2007; Shoham et al. 2022). Because previous studies investigating neural differences in DP have primarily focused on the core and extended face networks or on connectivity between pairs of regions, it is unclear whether interactions across a wider network of the brain, including regions outside the core and extended face network may also account for the difficulties evident in DP.

In this study, we used data-driven analyses to investigate differences in whole-brain functional and structural connectivity between individuals with DP and neurotypical Controls. Whole-brain connectivity approaches offer a comprehensive means to examine how neural responses are integrated across the brain and identify functional and structural alterations in brain organization that may not be evident when the analysis is restricted to specific pairs of regions (Martuzzi et al. 2011; Whitfield-Gabrieli and Nieto-Castanon 2012; Nieto 2022). Specifically, we conducted whole-brain analyses by quantifying the magnitude and pattern of functional connectivity between each voxel and all other voxels in the brain. A key aspect of our design is the comparison between functional connectivity patterns elicited during the viewing of face stimuli versus nonface stimuli (flowers), allowing us to assess the functional specificity of any connectivity differences observed in DP. Additionally, to determine whether potential functional connectivity alterations are coupled with disruptions in the underlying structural connectivity, we compared white matter connectivity between DPs and neurotypical Controls.

## Materials and methods

### Participants

Twenty-two DPs (4 males, M_age_ = 41.59, SD_age_ = 11.82) and 20 typical Controls (8 males, M_age_ = 34.10, SD_age_ = 12.62) participated in the study. DP participants were recruited through www.troublewithfaces.org. Diagnostic evidence for the presence of DP was collected using the PI20 questionnaire—a 20-item self-report measure of face recognition abilities (Shah et al. 2015) and the Cambridge Face Memory Test (CFMT)—an objective measure of face recognition (Duchaine and Nakayama 2006b). The use of convergent diagnostic evidence from self-report and objective computer-based measures of face recognition ability is thought to provide reliable identification of DP (Gray et al. 2017). The inclusion criteria for DP individuals was to score >2 SDs from the typical mean on the PI20 and <2SD on the CFMT. All DPs in the final sample scored at least 2.8 standard deviations above the typical mean on the PI20 and at least 2.3 standard deviations below typical mean on the CFMT. Supplementary Tables S1 and S2 show demographic and diagnostic information for individual DP and Control participants. Typical Controls were recruited from the local community. As expected, the DPs and Controls differed significantly in terms of their PI20 (M_DP_ = 78.36, SD = 7.20, M_control_ = 36.55, SD = 8.29, t(40) = 17.50, *P* < 0.001) and CFMT scores (M_DP_ = 55.43, SD = 6.99, M_control_ = 81.04, SD = 8.89, t(40) = 10.43, *P* < 0.001). The groups did not differ significantly in terms of their age, t(40) = 1.99, *P* > 0.05, or proportion of males [*X^2^*_(1)_ = 2.44, *P* > 0.05].

All participants were over 18 yr old, had normal or corrected-to-normal vision and had no history of psychiatric, neurological conditions, and autism. All participants provided written informed consent and the experiment was approved by the York Neuroimaging Centre (YNiC) Ethics Committee.

### Experimental design and statistical analysis

**Fig. 1:**
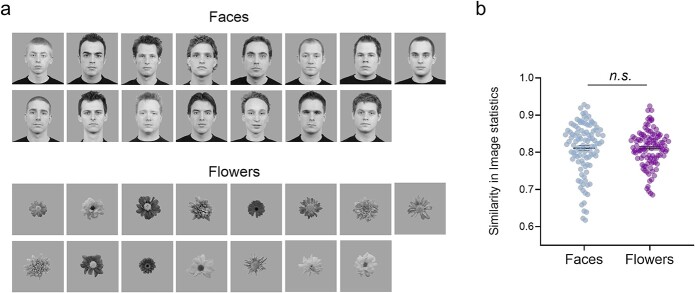
(a) Fifteen face and flower images used in the fMRI experiment. (b) The average similarity between exemplars was similar for faces and flowers. Each dot represents the similarity in image statistics between a pair of images measured using correlation.

Fifteen different face images and 15 flower images were used for the face and flower scans, respectively (Fig. 1a). All images were gray-scale on a mid-gray background with a resolution of 400 × 400 pixels. Face images were taken from the Radboud face database (Langner et al. 2010). All faces were front-facing white males, had neutral facial expression and were unknown to the participants. All flowers were from the *Asteraceae* family and were taken from the SOLID database (Frank et al. 2020). Examples of face and flower images are shown in Fig. 1a. Images were presented using a blocked design. Within each scan, each unique face/flower image was presented for 4 blocks (in pseudorandomized order such that all 15 identities are presented before repeating an identity). Within a block, individual faces/flowers were repeated 4 times for 600 ms, with a 200 ms inter-stimulus-interval. This was followed by an inter-block interval lasting 6 s. In total, each of the unique face/flower identities were seen 16 times (4 presentation × 4 repetition within a block). The face scan was always presented before the flowers scan for all participants. To maintain attention during the scan, participants were required to press a button when the fixation cross changes from black to green, which occurred randomly 60 times throughout the scan. There was no significant difference between the accuracy of responses to fixation cross changes between the face (mean = 94.8%, SEM = 0.15%) and flower (mean = 95.5%, SEM = 0.14%) scans (t(118) = 0.37, *P* > 0.05). There was also no significant difference in the reaction times between the face (mean = 567 ms, SEM = 22 ms) and flowers (mean = 558 ms, SEM = 21 ms) scans (t(118) = 1.06, *P* > 0.05). The total length of each scan was 9 min. In choosing the number and repetition frequency of images, we aimed for control participants to achieve visual familiarity to the face identities in order to capture functional connectivity between regions that are involved in this process and find neural differences in DPs who are behaviorally impaired at achieving familiarity for faces.

To ensure that the face and flower exemplars were comparable in low-level similarity, we measured their low-level properties using GIST (Torralba and Oliva 2001). The GIST descriptor measures the spectral and spatial properties of an image. Each image was spatially divided into 64 (8 × 8) locations. The GIST descriptor calculates low-level properties by convolving the image with 32 Gabor filters at 4 spatial scales, each with 8 orientations, producing 32 feature maps for each of the 64 spatial locations. This produces a total of 2,048 values describing the low-level properties of each image. First, we measured the GIST for all 15 face and flower images. Next, we correlated the resulting vector of each face with the other 14 faces and each flower with the other 14 flowers, producing 105 values describing the similarity in low-level properties between each pair of images for the face and flower categories. Next, we compared the similarity between faces and the similarity between flowers and found there was no significant difference between the face and flower categories, t(208) = 0.03, *P* = 0.979 (Fig. 1b).

### fMRI data acquisition

fMRI data were acquired with a GE 3 T HD Excite MRI scanner at YNiC (University of York) using an 8-channel phased-array, head-dedicated gradient insert coil tuned to 127.4 Hz. A gradient echo-planar imaging (EPI) was used to collect data from 38 contiguous slices (TR = 3000 ms, TE = 25 ms, FOV = 288 × 288 mm, matrix size = 128 × 128, slice thickness = 3 mm). High-resolution T1-weighted anatomical images were acquired (TR = 2300 ms, TE = 2.26 ms, FOV = 256 × 256 mm, matrix size = 256 × 256, 1 mm^3^).

### Functional connectivity analysis

Preprocessing and analysis of functional connectivity was carried out with the CONN toolbox (https://web.conn-toolbox.org/fmri-methods; Whitfield-Gabrieli and Nieto-Castanon 2012) using the default preprocessing pipeline, which includes realignment and unwrapping, slice-timing correction, outlier detection, structural and functional segmentation and normalization and spatial smoothing (8 mm Gaussian kernel). Functional and anatomical data were normalized into standard Montreal Neurological Institute (MNI) space and segmented into gray matter, white matter, and CSF tissue classes using the CONN in-built statistical parametric mapping 12 (SPM12) unified segmentation and normalization procedure (Ashburner and Friston 2005). Functional connectivity analyses used the time series from the gray matter voxels only. Next, functional data were denoised by applying the default denoising pipeline. This included the anatomical component-based noise correction aCompCor (Behzadi et al. 2007) by modeling out the sources of noise from white matter, gray matter, and cerebrospinal fluid as nuisance parameters within the first-level general linear model (GLM). Scrubbing, motion regression (12 regressors: 6 motion parameters + 6 first-order temporal derivatives), and temporal band-pass filtering (0.008 to 0.09 Hz) were applied. Scrubbing is a process in which a number of contaminated volumes (change in bold signal attributed to head motion) are regressed out at the denoising state of CONN. There were no significant differences in head movement between the groups in the face [t(40) = 0.70, *P* = 0.488] or flowers [t(40) = 1.00, *P* = 0.323] scans. One Control participant was excluded from further analysis for excessive head motion (Supplementary Fig. S1).

To measure global connectivity, a voxel-based functional intrinsic connectivity contrast (ICC) was used to measure the strength of functional connectivity between each voxel and every other voxel during the full duration of each scan (Martuzzi et al. 2011). The strength of connectivity between each voxel and the average of the rest of the voxels in the brain (root mean square of correlation coefficient values between a voxel and the rest of the brain) provided a measure of the functional centrality at each voxel. Voxels with higher connections to the rest of the brain are regarded as functionally central and thus more globally connected. Individual-level ICCs were calculated and combined into Control and DP group-level analyses. To determine if there were voxels that showed a different level of connectivity in DPs, we performed a group contrast using the CONN in-built cluster-level inference based on Gaussian Random Field theory with voxel-thresholding at *P* < 0.005 and a cluster-size false discovery rate (FDR)-corrected at *P* < 0.05. We chose a voxel-threshold of *P* < 0.005 based on the consideration that a voxel will be differently (less or more) globally connected in DPs only if it is significantly less or more connected to a sufficiently large number of other voxels. As such, if an area had decreased/increased connectivity to only one other area, this might not be sufficient to result in reduced global connectivity; thus, in choosing *P* < 0.005, we aimed to strike a balance between the most conservative threshold and false-positives.

Changes in connectivity may not only be based on overall changes in the magnitude of connectivity but also on the pattern of connectivity. That is, the connectivity of a particular area does not necessarily need to be lower, but the pattern of functional projections might be different. To address this, we used functional connectivity multivariate pattern analysis (fc-MVPA). This analysis computes the connectivity patterns characterizing the connectivity between each voxel and the rest of the brain (Nieto 2022). The fc-MVPA calculates functional connectivity between each voxel and the rest of the voxels in the brain for each subject and computes a reduced set of eigenpattern (principal component) scores best characterizing relevant spatial features of these maps across subjects. In the current analysis, we used the first 4 components. Cumulatively, the first 4 components explained 90.02 and 89.70% of the variance in connectivity profiles across all participants in the faces and flowers scans, respectively. Once each subject’s functional connectivity profiles are represented in terms of the 4 lower-dimensional eigenpattern scores, group-level functional connectivity analyses are computed by entering these scores into a standard GLM that evaluates the hypothesis that there will be a group difference (Control vs DP) in the connectivity pattern of a given voxel using the likelihood-ratio test. This procedure is repeated for each voxel sequentially, constructing a statistical parametric map across the entire brain. A group contrast was used to identify any regions with different patterns of connectivity in DPs compared to Controls (voxel-threshold *P* < 0.005, cluster-size FDR-corrected *P* < 0.05).

### Diffusion MRI (dMRI) data acquisition and preprocessing

We investigated structural connectivity across the brain using diffusion tensor imaging (DTI). DTI is an MRI technique that measures the directionality of water molecule diffusion to determine the structure of white matter tracts in the brain (Basser et al. 2000). Diffusion-weighted MRI (dMRI) data were acquired with a GE 3 T HD Excite MRI scanner with an 8 channel whole head high-resolution brain array. Two dMRI scans were acquired, with opposing phase encoding directions. The first dMRI scan lasted ~9 min with posterior-to-anterior phase encoding direction. A single-shot pulsed gradient spin-echo EPI sequence was used with the following parameters: b = 1,000 s/mm^2^, 25 unique diffusion directions, 60 slices, FOV = 192 mm, TR = 12 s, TE = 88.5 ms (minimum full), voxel size = 2 × 2 × 2 mm^3^, matrix size = 96 × 96, flip angle = 90°. Three volumes without diffusion weighting (b0) were acquired at the start of the scan. The second dMRI scan was ~4.5 min with anterior-to-posterior phase encoding direction and was used to correct distortion and had only 12 diffusion directions. All other scan parameters were the same as the first dMRI scan. Data were split across time and the first 3 baseline volumes with no diffusion were extracted. The b0 volumes were merged across the 2 scans to estimate the amount of susceptibility-induced distortion and Topup (Andersson et al. 2003) was used to correct it by applying the distortion field to the scans and combining them together. Nonbrain tissue removal was applied using BET (Smith 2002).

The diffusion data were reconstructed using generalized q-sampling imaging (Yeh et al. 2010) with a diffusion sampling length ratio of 1.25. The tensor metrics were calculated using DWI with a b-value of 1000 s/mm^2^. Generalized q-sampling imaging first reconstructs diffusion-weighted images in native space and computes the quantitative anisotropy (QA) in each voxel. These QA values are used to warp the brain to a template QA volume in MNI space using the SPM nonlinear registration algorithm. Once in MNI space, spin density functions were again reconstructed with a mean diffusion distance of 1.25 mm. A deterministic fiber tracking algorithm (Yeh et al. 2013) was used with augmented tracking strategies (Yeh 2020) to improve reproducibility. The anisotropy threshold was randomly selected. The angular threshold was randomly selected from 15° to 90°. The step size was randomly selected from 0.5 voxels to 1.5 voxels. Tracks with lengths shorter than 30 or longer than 300 mm were discarded. The Harvard-Oxford parcellation was registered to the *b*0 volume from each subject’s diffusion data. A total of 1,000,000 seeds were placed. Whole-brain tractography was conducted using DSI Studio (http://dsi-studio.labsolver.org).

### Connectome construction and topological measures

The connectome model was constructed by parcellating the whole-brain tracts with 96 cortical regions derived from the Harvard-Oxford atlas. The connectivity matrix was calculated by using the number of tracts connecting each pair of anatomical regions. The weighted connectivity matrix for each participant was thresholded at 10% of the overall count sum to preserve the strongest anatomical connection and then binarized. At this threshold, mean densities of 7.48 and 7.12% were calculated across connectomes in Controls and DPs, respectively.

A DTI-derived brain network for each subject can be described as a graph with set of nodes representing regions of brain and edges that form the white matter connections between the nodes. Topological measures of brain networks can be represented in a number of ways, all of which capture different features of connectivity. In the current study, we used the graph measures of degree at the whole network and nodal levels to ensure the structural connectome analysis is consistent with the global functional connectivity analysis. The degree represents the number of edges connected to a node (brain region). The degree of each node measures its integration with the broader network (global connectivity to the rest of the brain regions). The degree of a node $i$ is given by:


$$ k(i)=\sum_{j\in N}a\left(i,j\right) $$

where $a\left(i,j\right)$ is the connection status between the pair of regions $i$ and $j$.

## Results

### Analysis of functional connectivity

**Fig. 2:**
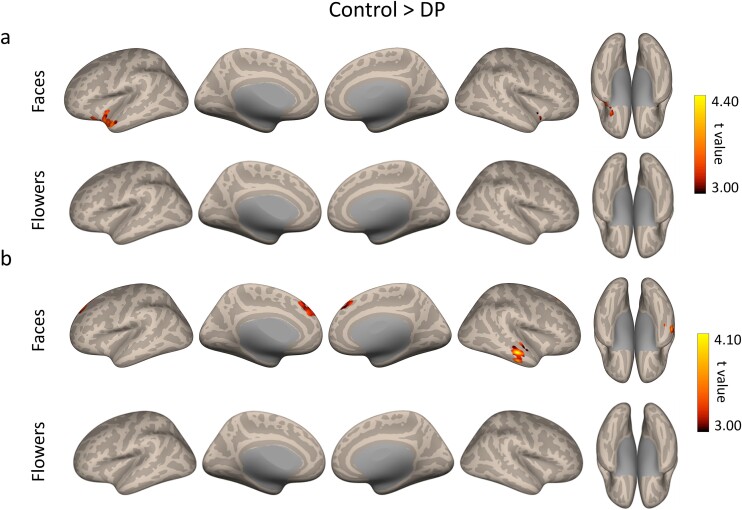
(a) Analysis of global functional connectivity. Difference in whole-brain global connectivity between Control and DP groups for the faces and flowers scans. The LTP showed a significant difference in global connectivity between Controls and DPs. Differences in connectivity were evident when viewing faces but not flowers. (b) Analysis of seed-based functional connectivity. Regions showing lower connectivity between the LTP (seed) in DPs compared to control participants for the faces and flowers scans. Reduced connectivity was evident in the medial prefrontal cortex and within regions of the temporal lobe when viewing faces but not flowers.

We measured whole-brain global connectivity differences between Controls and DPs during the face and flower scans. Areas with significantly lower global connectivity in DP compared to Controls when viewing faces are shown in Fig. 2a, Table 1 and Supplementary Fig. S2. The overlap of the areas with cortical and subcortical parcellations from the Harvard-Oxford atlas is shown in Supplementary Table S2. The majority of voxels with lower global connectivity in DPs fell within the left temporal pole (LTP). The peak location of these voxels on the dorsal surface of the temporal pole is distinct from location of the anterior temporal face patch, which appears on the ventral surface of the temporal lobe (Rajimehr et al. 2009). We also found lower global connectivity in voxels within the right putamen, left and right amygdala, left orbitofrontal cortex and left and right insula. In contrast, there was no difference in global connectivity between Controls and DPs in any region when viewing flowers (Fig. 2a), suggesting a selective reduction in global connectivity during face processing for DP. We found no significant voxels that showed greater global functional connectivity for DPs compared to Controls.

**Table 1:** Peak MNI coordinates, *P* value (FDR-corrected), top anatomical region (region containing the most overlapping significant voxels), and number of overlapping voxels of the significant clusters from the ICC analysis, LTP seed-based analysis, and MVPA faces and flower analyses.

	**Peak coordinates**			***P* value**	**Top anatomical region**	**Overlapping voxels**
	**x**	**y**	**z**	**(FDR)**		
**ICC faces**	−44	12	−26	0.003	LTP	187
	33	0	−12	0.004	Right putamen	106
**LTP (seed)**	12	36	48	0.007	Left frontal pole	190
	50	−16	−22	0.028	Right posterior middle temporal gyrus	219
**MVPA faces**	−60	−58	4	<0.001	Left middle temporal gyrus	225
	38	−54	−12	0.005	Right temporal occipital fusiform cortex	109
	6	−74	32	0.009	Precuneus cortex	127
	−40	−8	−28	0.016	LTP	69
	−2	−36	−54	0.035	Brain stem	111
**MVPA flowers**	8	−64	30	0.023	Precuneus cortex	130
	18	−12	−10	0.023	Brain stem	25
	−38	−64	−24	0.023	Cerebellum	83
	22	−44	−8	0.042	Lingual gyrus	97

To determine which functional connections are lower in DPs, we performed a seed-based analysis. We focused on the LTP as this region showed the greatest difference in global functional connectivity. Seed-based connectivity with the other significant regions (right putamen and left and right amygdala) are shown in Supplementary Fig. S3 and Supplementary Table S3. The reference time series for the LTP was obtained by averaging the time series of all voxels within the seed region. A seed-to-voxel functional connectivity was calculated for Controls and DPs, and then a group contrast was carried out (voxel-threshold *P* < 0.005, cluster-size FDR-corrected *P* < 0.05). In DPs, the LTP had weaker contralateral connections to the anterior regions of the superior, middle, and inferior temporal gyri, as well as weaker bilateral connections to the medial prefrontal cortex when viewing faces (Fig. 2b, Table 1 and Supplementary Fig. S4). Supplementary Table S4 shows the full list of Harvard-Oxford parcellations that overlap with the significant voxels. Next, we asked whether the seed-based reductions in connectivity to the LTP were specific for faces by repeating the seed-based connectivity analysis while participants viewed flowers. When viewing flower images, we found that the LTP showed no difference in connectivity in DPs compared to Controls (Fig. 2b). Again, this suggests that the change in connectivity with the LTP is specific to face processing.

### Multivariate analysis of functional connectivity

**Fig. 3:**
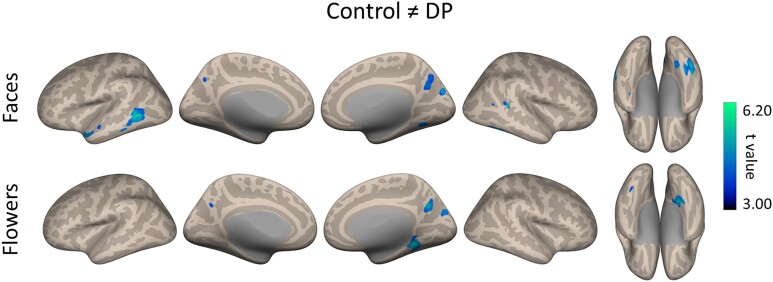
Multivariate analysis of functional connectivity. Differences in the whole-brain global functional connectivity patterns between DPs and Controls when viewing faces and flowers. Different patterns of global connectivity were evident in posterior and anterior regions of the middle temporal gyrus, the posterior superior temporal gyrus, the fusiform gyrus and the precuneus when viewing faces. A different pattern of change was evident when viewing flowers.

The global connectivity analysis aims to uncover *quantitative* group differences in the strength of connectivity. However, functional connectivity may also differ *qualitatively*; i.e. different patterns of connectivity may be seen in DPs and Controls. That is, the strength of connectivity of a particular voxel does not necessarily need to be lower or higher in one group, but the pattern of functional projections might be different. To address this possibility, we performed a multivariate analysis of the patterns of functional connectivity (fc-MVPA). The multivariate connectivity analysis addresses this by considering separately for each voxel the entire multivariate pattern of functional connections between this voxel and the rest of the brain. In particular, for any individual hypothesis (e.g. DP ≠ Control), the multivariate connectivity analysis will produce a statistical parametric map evaluating that hypothesis separately at each individual voxel. Voxels with a significantly different pattern of functional connections in DP are shown in Fig. 3 and Table 1 (Supplementary Tables S5 and S6, Figs. S5 and S6).

Again, we found that voxels in the LTP showed a different pattern of connectivity in DPs compared to Controls when viewing faces. We also found different cortical patterns of connectivity in the posterior and anterior regions of the temporal lobe, namely in the left middle temporal gyrus and right fusiform cortex. These regions were not found in a corresponding analysis of patterns of connectivity when viewing flowers. Posterior regions (precuneus and lingual gyrus) also showed a different pattern of connectivity in DPs compared to Controls. However, it is difficult to assess face-selective nature of these effects as differences between DPs and Controls were also evident in similar regions when viewing flowers.

### Whole-brain structural connectivity

We first calculated global structural connectivity across the whole brain. We measured the number of connections (edges) to each region (node) at the whole network level to give a value for the degree of connectivity. There was no overall difference in the degree (number of structural connections) between Controls (M = 6.73, SD = 0.92) and DPs (M = 6.41, SD = 0.92) across the whole brain (t(39) = 1.10, *P* = 0.274). Next, we determined if there were group differences in the degree of connectivity across different regions from the Harvard-Oxford atlas (Supplementary Fig. S7). Although the majority of regions had a higher degree of connectivity in Controls, none of the regions were significantly different at *P* < 0.05 (FDR-corrected for multiple-comparisons). The structural connectivity did not reveal any voxels that showed greater structural connectivity for DPs compared to Controls.

**Fig. 4:**
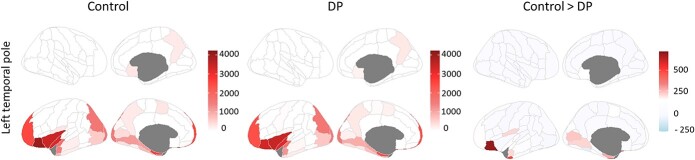
Structural connectivity with the temporal pole. Number of fibers linking the LTP and the rest of the brain regions in Controls, DPs, and Control > DP contrast. DPs had fewer connections with the anterior inferior temporal gyrus, the orbitofrontal cortex, and the anterior temporal fusiform cortex.

The degree of a region measures the overall connectivity of that region to the rest of the regions in the brain. However, as DPs have selective face recognition deficits, it is possible that DPs’ poor recognition abilities are associated with reductions in connectivity to a limited number of regions. To explore this possibility, we carried out a seed-based structural connectivity analysis in which we measured the number of connections between the seed and each of the other brain regions. We chose the LTP as a seed based on the fact that it showed consistently reduced connectivity in both functional connectivity analyses and allowed us to explore whether the observed reduction in strength and pattern of functional connectivity is mirrored by structural underconnectivity. We asked whether the number of fibers between the LTP and other brain regions is lower in DPs. Three regions showed significantly lower connections to the LTP in DPs: the left anterior inferior temporal gyrus (*P* = 0.004, FDR-corrected), the left orbitofrontal cortex (*P* < 0.0001, FDR-corrected), and the left anterior temporal fusiform cortex, (*P* = 0.031, FDR-corrected) (Fig. 4).

**Fig. 5:**
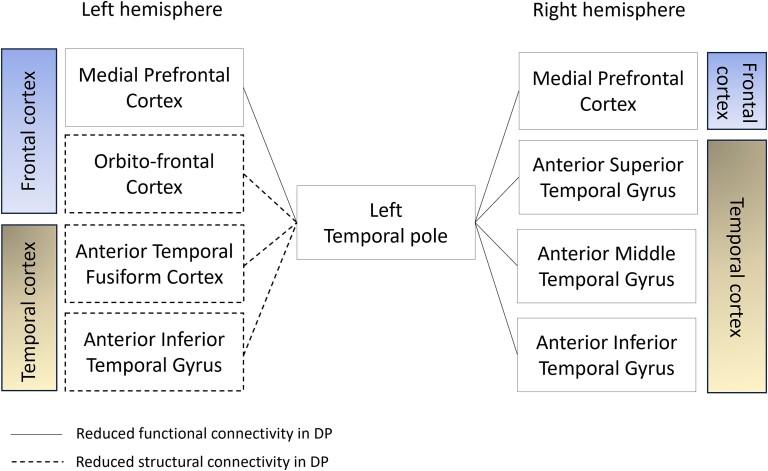
Schematic representations of the regions with reduced functional and structural connectivity with the LTP in DP.

A consistent finding across the functional and structural connectivity analyses has been the lower connectivity of the LTP in DP. A summary of regions with reduced functional and structural connectivity to the LTP in DP found across the current functional and structural seed-based analyses are presented on Fig. 5.

## Discussion

We used data-driven analyses to explore differences in whole-brain functional and structural connectivity in individuals with DP. Our findings revealed significant global changes in both the strength and pattern of functional brain connectivity in DPs compared to neurotypical Controls. These changes were predominantly driven by the LTP, which showed both weaker connections and atypical connectivity patterns with the rest of the brain. These connectivity differences were specific to face stimuli and were not observed when participants viewed nonface stimuli, such as flowers. These results suggest that typical face recognition depends on the integration of information across many brain regions and that disruptions to the integration of information can give rise to the deficits characteristic of DP.

Previous studies that have explored the neural basis of DP have predominantly focussed on face-selective regions of the human brain (Duchaine and Nakayama 2006a; Avidan and Behrmann 2014). However, recognizing and appropriately interacting with familiar individuals requires the integration of visual information with nonvisual episodic, semantic, and affective information (Gobbini and Haxby 2007; Shoham et al. 2022). To address whether more global deficits in integrating information might explain the deficits in DP, we measured whole-brain connectivity across the whole brain. Our analysis revealed global changes in functional connectivity in the LTP of individuals with DP, consistent with the region’s role in the recognition of identity. Activity in the anterior temporal cortex increases when viewing familiar faces (Gorno-Tempini et al. 1998; Leveroni et al. 2000; Sugiura et al. 2001; Gobbini and Haxby 2007; Collins and Olson 2014) and this region can support fine-grained perceptual discrimination of face identities (Kriegeskorte et al. 2007; Anzellotti et al. 2014; Yang et al. 2016). The temporal pole has also been proposed to serve a critical link between perception and memory systems (Collins et al. 2016). Our results align with previous findings showing that deficits in connectivity with the anterior temporal lobe are linked to face recognition ability in DP and neurotypical populations (Rosenthal et al. 2017; Levakov et al. 2023).

Seed-based connectivity analyses showed that the observed global reduction in connectivity of the LTP in DPs reflected interhemispheric connections with regions in the right temporal lobe. Previous research has established the significance of interhemispheric connectivity, demonstrating that connectivity between corresponding face-processing regions across the hemispheres is greater than within the same hemisphere (Davies-Thompson and Andrews 2012; Zhen et al. 2013). Interhemispheric connectivity has been linked to the integration of perception with memory formation. Notably, an increase in interhemispheric functional connectivity following incidental learning of faces has been associated with successful memory outcomes (Geiger et al. 2016). Furthermore, a recent study by Levakov et al. (2023) found that the connectivity between left anterior temporal lobe and the right temporal lobe predicted face recognition performance. Collectively, these results underscore the critical role of interhemispheric integration in supporting face recognition abilities.

Reduced connectivity was also evident between the LTP and medial prefrontal cortex. Personally familiar faces compared elicit increased responses in the medial prefrontal cortex compared to visually familiar faces (Gobbini et al. 2004; Leibenluft et al. 2004; di Oleggio et al. 2021). This region is involved in associating person traits with faces (Gobbini and Haxby 2007; Ramon and Gobbini 2018). The observed reduction in connectivity between the LTP and the medial prefrontal cortex suggests that typical face recognition relies on the integration of information across these regions. The fact that alterations in connectivity were present during the viewing of unfamiliar faces indicates that DPs’ deficits involve an impaired ability to acquire perceptual familiarity. Future research directly comparing connectivity patterns between familiar and unfamiliar faces will be important for elucidating the nature of the deficit in DP.

When we get to know a person, we develop associations between their face and the affective response that is elicited. Previous studies have shown that the regions involved in affective processing and making emotional responses include the amygdala, insula, orbitofrontal cortex, and regions of the striatal reward system (Gobbini and Haxby 2007; Ramon and Gobbini 2018). We found reduced global connectivity in DPs in the amygdala, putamen, insula, and orbitofrontal cortex—regions involved in affective processing and making emotional responses (Gobbini and Haxby 2007; Ramon and Gobbini 2018).

Changes in connectivity may not only reflect overall changes in the magnitude of connectivity but may also involve differences in the pattern of connectivity. That is, the connectivity of a particular area does not necessarily need to be lower, but the pattern of functional projections might be different. The aim of our MVPA connectivity analysis was to transcend the traditional approaches and investigate changes in the pattern of connectivity. Our analysis revealed altered patterns of global connectivity in the LTP. In addition, we also identified altered connectivity patterns in the middle temporal cortex, the posterior superior temporal cortex, and the fusiform cortex. Areas within these regions are typically associated with selectivity for faces relative to nonface objects and form the core face-selective network (Kanwisher et al. 1997; Haxby et al. 2000; Kanwisher 2010). These findings suggest that disruptions in the functional integration of these key regions may contribute to the face recognition deficits observed in DP.

A fundamental aspect of familiarity involves the acquisition of biographical and episodic memories associated with individuals. Regions implicated in episodic memories include the medial temporal lobe and the precuneus (Squire et al. 2004; Trimble and Cavanna 2008). Previous research has demonstrated that familiar faces elicit heightened responses in the precuneus (Leveroni et al. 2000; Gobbini et al. 2004; Visconti di Oleggio Castello et al. 2021). Our analysis revealed the pattern of functional connectivity between the precuneus and the rest of the brain differed in individuals with DP compared to neurotypical Controls during face viewing. However, we also found a difference in the pattern of connectivity while viewing flowers. This suggests a more general deficit in processing in DP and may be linked to the impairments in recognizing various nonface categories frequently reported in individuals with DP (Behrmann et al. 2005; Biotti et al. 2017; Geskin and Behrmann 2018).

To evaluate the selectivity of the effects observed with faces, we compared connectivity while viewing flowers. Our findings indicate that within-exemplar variation of image properties was comparable between face and flower stimuli. Nevertheless, inherent differences in complexity and visual properties exist between faces and flowers. A potential limitation of using flowers is that they may lack the complexity of visual properties found in faces. If the more complex image properties of faces, relative to flowers, were driving our effects, it would imply that DPs have a deficit with more complex images. However, this is not supported by the literature, with DPs showing normal recognition performance with complex nonface images (Duchaine et al. 2004; Fry et al. 2020). Furthermore, the most consistent effect throughout our analyses is the difference in connectivity of the LTP—a region not typically associated with image complexity. Given the established role of the anterior temporal pole in high-level perceptual and mnemonic representations, along with the established face recognition deficit in DP, our results argue against an explanation based on differences in image complexity between the categories. Future studies examining DP could benefit from comparing connectivity using a wider range of faces and objects that vary along different perceptual and conceptual properties.

The observed differences in functional connectivity could reflect alterations in functional connectivity or be driven by changes in the signal strength of the seed region. Disentangling these effects is inherently challenging, as functional connectivity is predicated on the similarity of neural signals across regions. A reduction in signal amplitude within the temporal pole could, in theory, influence inter-regional correlations, potentially allowing noise to dominate the connectivity estimates. Under such circumstances, one might anticipate a more generalized disruption, wherein connectivity between the LTP and a broad array of brain regions would be uniformly affected. Contrary to this expectation, our findings demonstrate that the connectivity deficit is predominantly driven by specific regions of the brain. Moreover, this effect is not evident when viewing flowers, suggesting that the observed changes are not simply a by-product of diminished signal in the seed region but likely reflect targeted disruptions in the neural networks implicated in face processing.

In addition to our functional connectivity analyses, we also investigated global structural connectivity. Our results indicated that the overall structural brain organization was similar between individuals with DP and neurotypical Controls, suggesting that DP is characterized by typical whole-brain structural organization but atypical functional organization. Previous studies have shown that structural connectivity can only explain 50% of the variance in functional connectivity (Honey et al. 2009), indicating that a significant portion of functional connectivity cannot be directly attributed to underlying structural connections. For example, Wang et al. (2020) functionally defined face-specific network of areas and measured the similarity of functional and structural connections between these areas but only found a weak to moderate correlation. Given the large gap in correspondence between structural and functional connectivity, a critical question concerns which connectivity measure has a better predictive power for cognitive abilities and behavior. While numerous studies have independently linked functional (Wang et al. 2009; Seo et al. 2013; Qian et al. 2019) and structural (Contreras et al. 2015; Bathelt et al. 2019) whole-brain connectivity to cognitive variability, relatively few studies have directly compared them in predicting cognition. Consistent with our current results, these studies demonstrated that whole-brain functional connectivity is a better predictor than structural connectivity of a range of cognitive abilities (Dhamala et al. 2021; Ooi et al. 2022). Our results contribute to a growing literature by showing that deficits in face recognition in DP are better captured by whole-brain functional, rather than whole-brain structural connectivity. However, we did find local reductions in structural connectivity in DP. DPs had fewer projections between the LTP and left anterior temporal and orbitofrontal cortex. These connectivity reductions overlap with the uncinate fasciculus and ILF and align with previous reports of structural alterations in the temporal lobe in DP (Thomas et al. 2009; Grossi et al. 2014; Gomez et al. 2015; Song et al. 2015; Metoki et al. 2017).

In conclusion, our results provide compelling evidence that deficits in face recognition associated with DP are linked to global changes in the strength and pattern of functional connectivity. Our findings demonstrate that these connectivity changes are evident at a whole-brain level without restricting analyses to predefined face-selective regions. Changes in connectivity were particularly evident in the LTP. We found that these connectivity changes were specifically associated with viewing faces, indicating that the integration of information over a wider network of regions is necessary to support typical face recognition.

## Supplementary Material

CerCor-2023-00694-R1_supplementary_bhae435

## Data Availability

Raw fMRI and dMRI data are not publicly available due to research participants not providing consent to share their data publicly. Pre-processed data and code are publicly available at https://osf.io/7kf4u/.
