# Supplementary material for: Global changes in the pattern of connectivity in developmental prosopagnosia
Source: Cereb Cortex. 2024 Nov 7;34(11):bhae435. doi: 10.1093/cercor/bhae435 (PMC11546179; doi:10.1093/cercor/bhae435)
Supplement: CerCor-2023-00694-R1_supplementary_bhae435 [file cercor-2023-00694-r1_supplementary_bhae435.docx]

**Supplementary Information**

**Global changes in the pattern of connectivity in developmental prosopagnosia**

**Supplementary Table 1.** Demographic information and individual scores on the diagnostic tests used to validate developmental prosopagnosia, namely the 20-Item Prosopagnosia Index (PI20) and the Cambridge Face Memory Test (CFMT).

| Participant | Gender | Age | PI20 | CFMT | zPI20 | zCFMT |
| --- | --- | --- | --- | --- | --- | --- |
| DP1 | F | 41 | 68 | 59.72 | 3.30 | -2.84 |
| DP2 | F | 34 | 76 | 56.94 | 4.18 | -3.15 |
| DP3 | F | 38 | 90 | 47.22 | 5.71 | -4.24 |
| DP4 | F | 55 | 74 | 58.33 | 3.96 | -3.00 |
| DP5 | M | 46 | 86 | 58.33 | 5.27 | -3.00 |
| DP6 | F | 47 | 87 | 44.44 | 5.38 | -4.56 |
| DP7 | F | 22 | 69 | 52.77 | 3.41 | -3.62 |
| DP8 | F | 26 | 83 | 59.72 | 4.95 | -2.84 |
| DP9 | M | 38 | 76 | 62.5 | 4.18 | -2.53 |
| DP10 | F | 52 | 84 | 54.17 | 5.05 | -3.46 |
| DP11 | F | 31 | 80 | 52.78 | 4.62 | -3.62 |
| DP12 | F | 59 | 72 | 62.5 | 3.74 | -2.53 |
| DP13 | M | 68 | 64 | 48.6 | 2.86 | -4.09 |
| DP14 | F | 30 | 67 | 63.89 | 3.19 | -2.37 |
| DP15 | F | 30 | 84 | 45.83 | 5.05 | -4.40 |
| DP16 | F | 28 | 80 | 41.67 | 4.62 | -4.87 |
| DP17 | F | 49 | 81 | 63.89 | 4.73 | -2.37 |
| DP18 | F | 42 | 75 | 48.61 | 4.07 | -4.09 |
| DP19 | F | 49 | 86 | 62.5 | 5.27 | -2.53 |
| DP20 | F | 42 | 82 | 62.5 | 4.84 | -2.53 |
| DP21 | M | 54 | 84 | 51.39 | 5.05 | -3.78 |
| DP22 | F | 34 | 76 | 61.11 | 4.18 | -2.68 |
| **DP Mean** | | **41.59** | **78.36** | **55.43** |  |  |
| **DP SD** | | **11.82** | **7.19** | **6.99** |  |  |
| **Comparison Mean** | | **39.20** | **38.0** | **85.0** |  |  |
| **Comparison SD** | | **13.4** | **9.1** | **8.9** |  |  |

Nb. Comparison data (N = 54) for the PI20 and CFMT were taken from Biotti, Gray & Cook (2019).

**Supplementary Table 2.** Demographic information and individual scores on the 20-Item Prosopagnosia Index (PI20) and the Cambridge Face Memory Test (CFMT).

| Participant | Gender | Age | PI20 | CFMT |  |  |
| --- | --- | --- | --- | --- | --- | --- |
| Control 1 | F | 21 | 35 | 80.56 |  |  |
| Control 2 | M | 22 | 35 | 72.20 |  |  |
| Control 3 | M | 31 | 25 | 100 |  |  |
| Control 4 | F | 28 | 42 | 76.39 |  |  |
| Control 5 | F | 22 | 35 | 94.44 |  |  |
| Control 6 | F | 22 | 36 | 83.33 |  |  |
| Control 7 | M | 23 | 42 | 80.55 |  |  |
| Control 8 | F | 62 | 36 | 73.61 |  |  |
| Control 9 | M | 41 | 27 | 93.06 |  |  |
| Control 10 | F | 23 | 25 | 76.39 |  |  |
| Control 11 | F | 55 | 34 | 83.33 |  |  |
| Control 12 | M | 53 | 49 | 84.72 |  |  |
| Control 13 | M | 30 | 28 | 76.39 |  |  |
| Control 14 | F | 36 | 54 | 80.56 |  |  |
| Control 15 | F | 43 | 42 | 94.44 |  |  |
| Control 16 | M | 32 | 28 | 75 |  |  |
| Control 17 | M | 47 | 28 | 84.72 |  |  |
| Control 18 | F | 41 | 45 | 66.67 |  |  |
| Control 19 | F | 22 | 49 | 73.61 |  |  |
| Control 20 | F | 28 | 39 | 70.83 |  |  |
| **Control Mean** | | **34.10** | **36.55** | **81.04** |  |  |
| **Control SD** | | **12.62** | **8.29** | **8.89** |  |  |

**
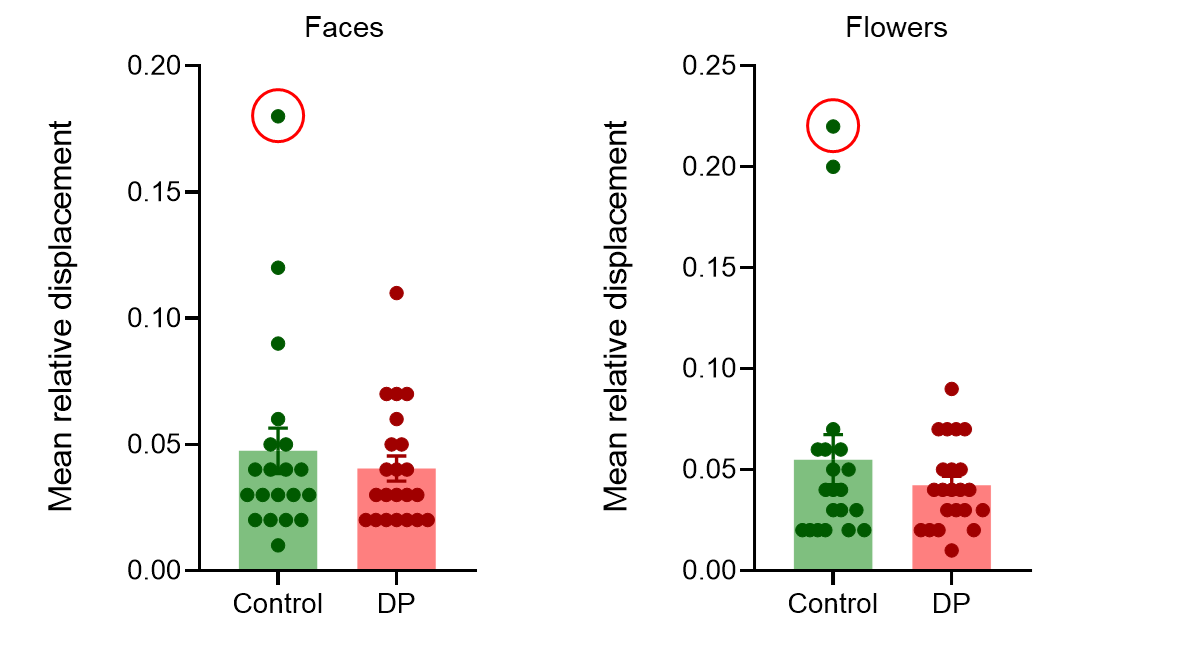
**

**Supplementary Figure 1.** Mean relative head displacement for Control and DP participants in the faces and flowers scans. There were no significant differences in head movement between the groups in the faces scan, t(40) = 0.70, p = 0.488, and flowers scan, t(40) = 1.00, p = 0.323. However, one control participant had a consistently high mean relative displacement across the 2 scans (Faces scan: M = 0.18, Flowers scan: M = 0.22) and was excluded from further analyses.

**Supplementary Table 2.** Overlap of voxels with higher global functional connectivity during face viewing in the Control>DP contrast with regions from the cortical and subcortical Harvard Oxford atlas (<https://neuroconductor.org/help/ggsegHO/>).

| \| Region \| Hemisphere \| Overlapping voxels \| \| --- \| --- \| --- \| \| Temporal pole \| left \| 187 voxels \| \| Putamen \| right \| 106 voxels \| \| Amygdala \| right \| 58 voxels \| \| Amygdala \| left \| 52 voxels \| \| Frontal Orbital Cortex \| left \| 43 voxels \| \| Insular Cortex \| left \| 29 voxels \| \| Pallidum \| right \| 27 voxels \| \| Pallidum \| left \| 12 voxels \| \| Hippocampus \| right \| 8 voxels \| \| Planum Polare \| left \| 5 voxels \| \| Insular Cortex \| right \| 2 voxels \| \| Putamen \| left \| 2 voxels \| |
| --- | --- | --- | --- | --- | --- | --- | --- | --- | --- | --- | --- | --- | --- | --- | --- | --- | --- | --- | --- | --- | --- | --- | --- | --- | --- | --- | --- | --- | --- | --- | --- | --- | --- | --- | --- | --- | --- | --- | --- |

**
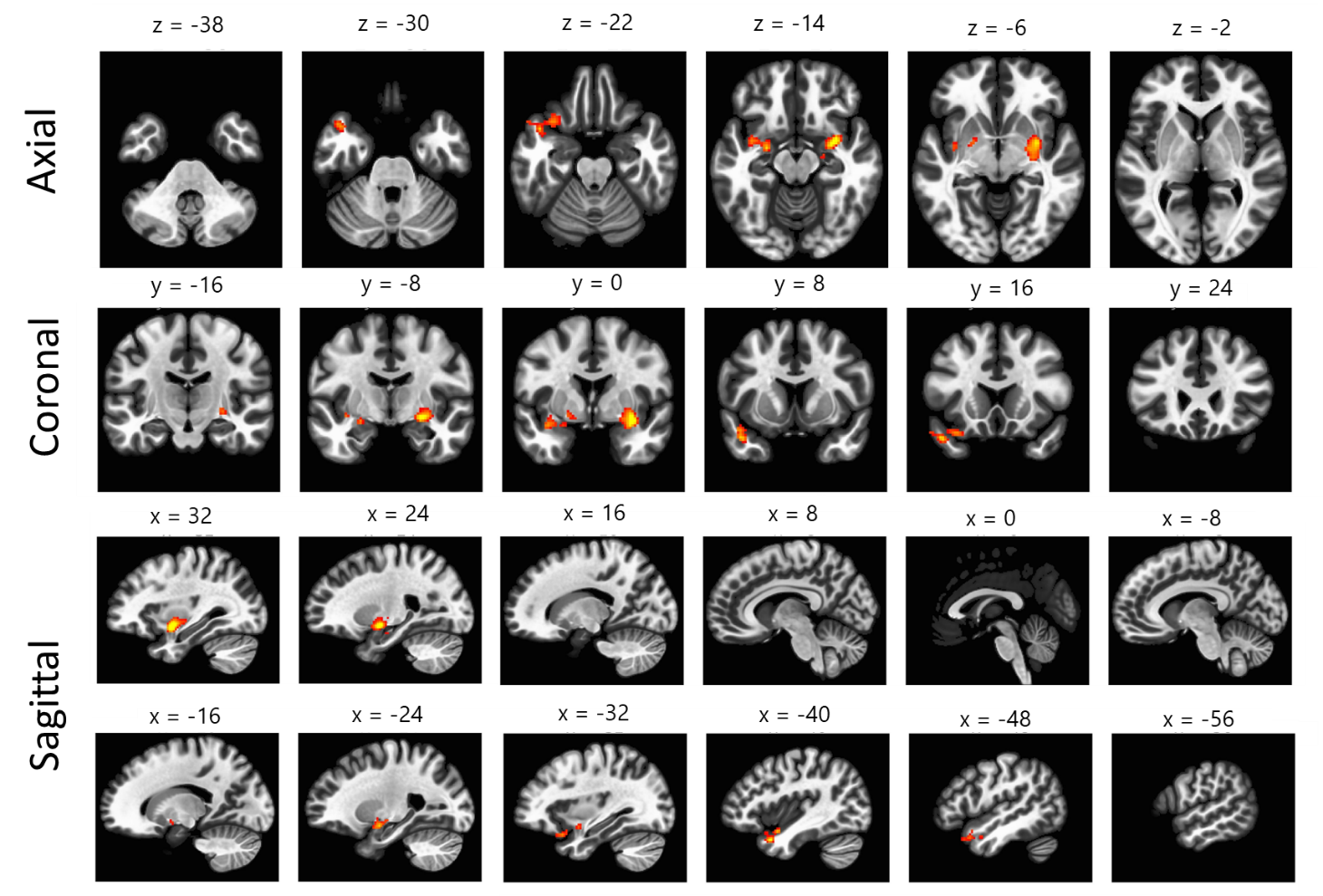
**

**Supplementary Figure 2.** Section-based presentation of the significant cortical and subcortical areas in whole-brain global connectivity between Control and DP groups for faces.


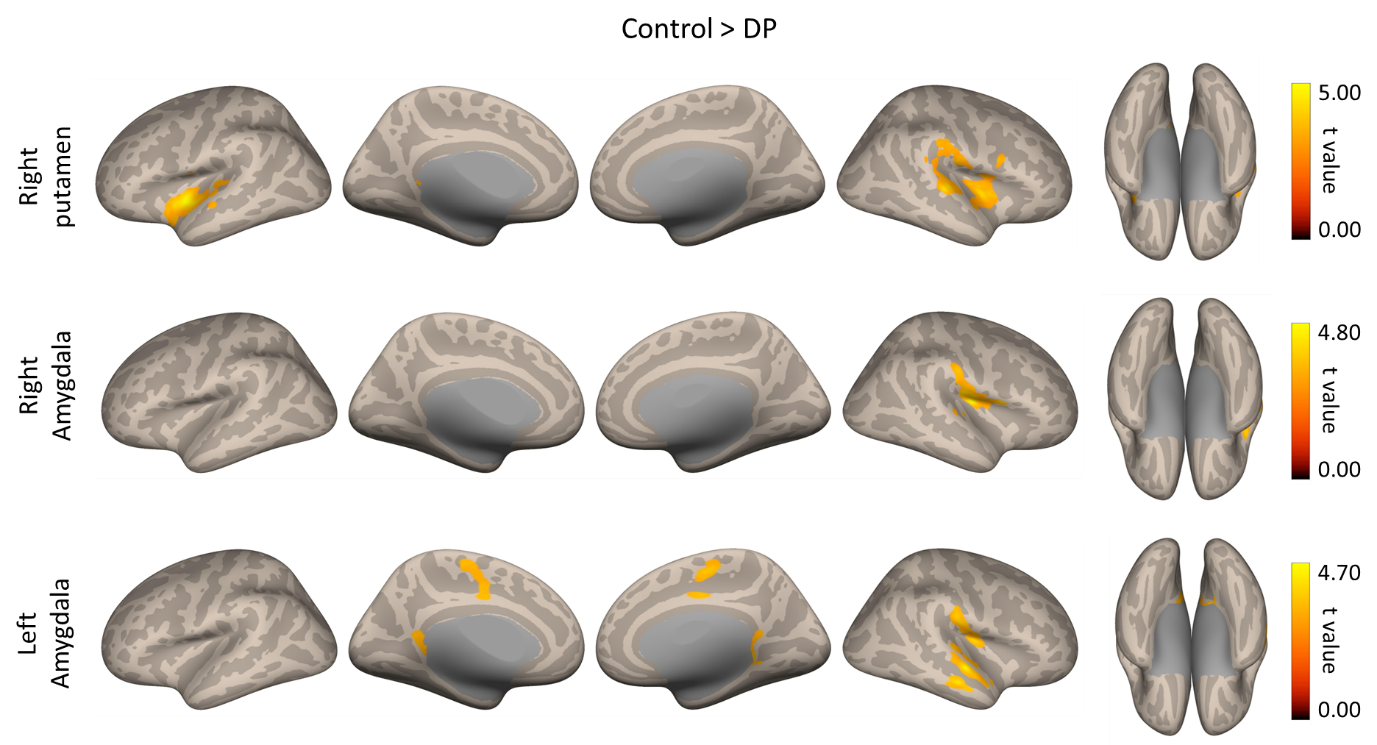


**Supplementary Figure 3.** Analysis of seed-based functional connectivity, voxel-thresholding at p<0.005 and a cluster-size FDR-corrected at p<0.05 Regions showing lower connectivity with the right putamen, right amygdala and left amygdala (seeds) in DPs compared to control participants during the faces scan.

**Supplementary** **Table 3.** Peak MNI coordinates, p value, top anatomical region (region containing the most overlapping significant voxels) and number of overlapping voxels of the clusters showing lower connectivity in DPs to the 3 seeds (right putamen, right amygdala and left amygdala.

|  | Peak coordinates | | |  |  |  |  |  |
| --- | --- | --- | --- | --- | --- | --- | --- | --- |
|  | x | y | z | p value (FDR) | Top anatomical region | |  | Overlapping voxels |
| Right putamen | -38 | -12 | -6 | 0.001 | left insular cortex | |  | 305 |
|  | 66 | -20 | 6 | 0.003 | right planum temporale | |  | 173 |
|  | 40 | -6 | -2 | 0.003 | right insular cortex | |  | 312 |
|  | -6 | -40 | 8 | 0.009 | left thalamus | |  | 137 |
| Right amygdala | 36 | -14 | 18 | 0.003 | right central opercular corte | |  | 320 |
| Left amygdala | 14 | -34 | 0 | 0.006 | right thalamus | |  | 190 |
|  | 56 | -10 | -8 | 0.006 | right posterior middle temporal gyrus | |  | 114 |
|  | 38 | -14 | 16 | 0.039 | right central opercular cortex | |  | 124 |
|  | 4 | -16 | 54 | 0.039 | anterior cingulate gyrus | |  | 64 |

**Supplementary Table 4.** Overlap of voxels with higher functional connectivity to the left temporal pole during face viewing in the Control>DP contrast with regions from the cortical and subcortical Harvard Oxford atlas (<https://neuroconductor.org/help/ggsegHO/>).

| Region | Hemisphere | Overlapping voxels |
| --- | --- | --- |
| Posterior Middle Temporal Gyrus | right | 219 voxels |
| Frontal Pole | left | 190 voxels |
| Superior Frontal Gyrus | left | 132 voxels |
| Superior Frontal Gyrus | right | 59 voxels |
| Frontal Pole | right | 37 voxels |
| Posterior Inferior Temporal Gyrus | right | 22 voxels |
| Anterior Middle Temporal Gyrus | right | 9 voxels |

**
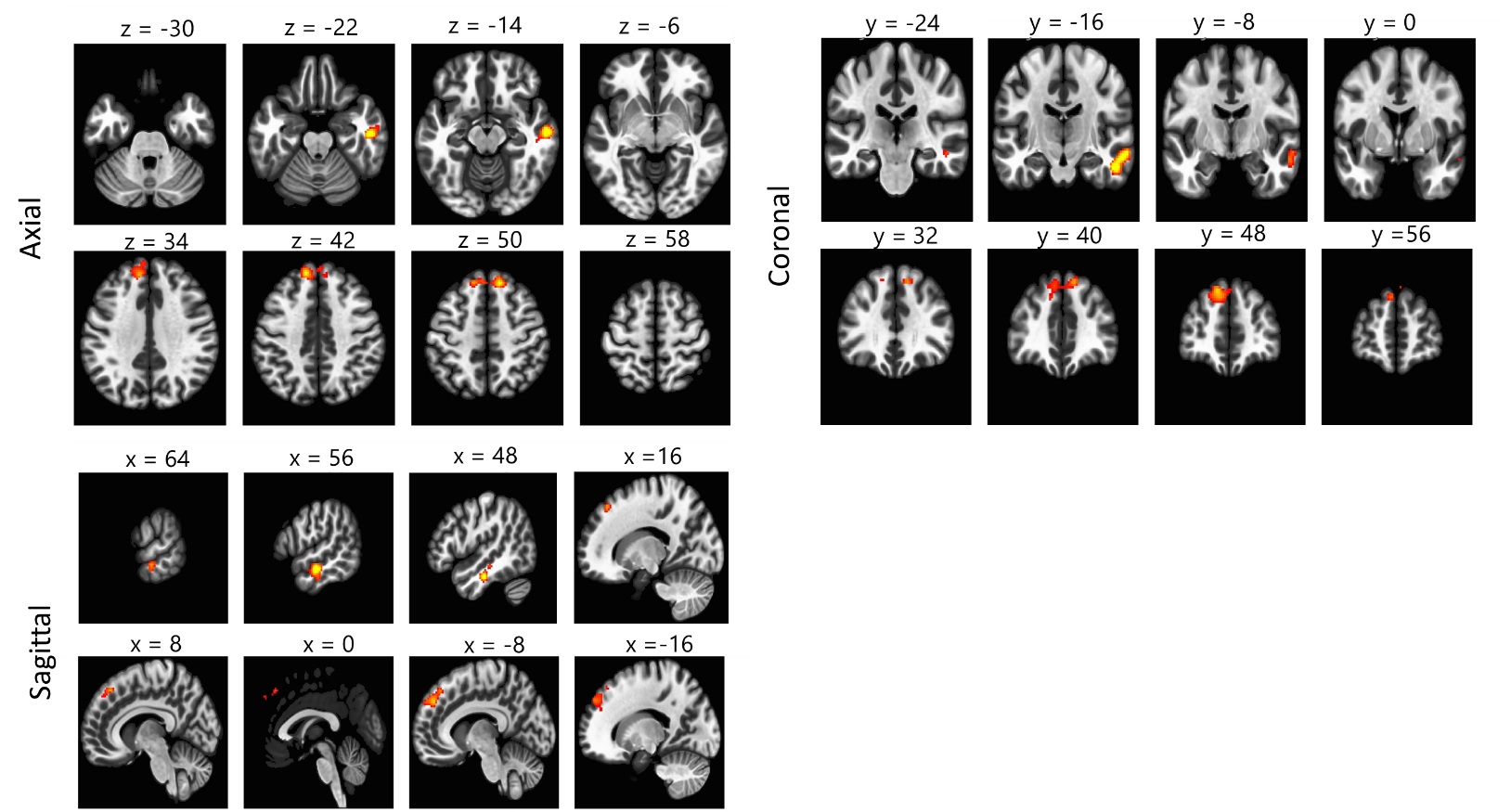
**

**Supplementary Figure 4.** Section-based presentation of the significant cortical and subcortical areas in the seed-based (left temporal pole) connectivity analysis between Control and DP groups for faces.

**Supplementary Table 5.** Multivariate analysis of functional connectivity. Overlap of voxels with different pattern of functional connectivity when viewing faces in the Control>DP contrast with regions from the cortical and subcortical Harvard Oxford atlas (<https://neuroconductor.org/help/ggsegHO/>).

| Region | Hemisphere | Overlapping voxels |
| --- | --- | --- |
| Temporo-occipital middle temporal gyrus | left | 225 voxels |
| Precuneus Cortex | both | 127 voxels |
| Brainstem | both | 111 voxels |
| Temporal Occipital Fusiform Cortex | right | 109 voxels |
| Temporal Pole | left | 69 voxels |
| Inferior Lateral Occipital Cortex | left | 69 voxels |
| Cuneal Cortex | right | 51 voxels |
| Lingual Gyrus | right | 13 voxels |
| Angular Gyrus | right | 7 voxels |
| Occipital Fusiform Gyrus | right | 7 voxels |
| Anterior Superior Temporal Gyrus | left | 4 voxels |
| Posterior Supramarginal Gyrus | right | 4 voxels |
| Temporo-occipital Inferior Temporal Gyrus | left | 3 voxels |
| Posterior Supramarginal Gyrus | left | 1 voxel |
| Anterior Temporal Fusiform Cortex | left | 1 voxel |

**
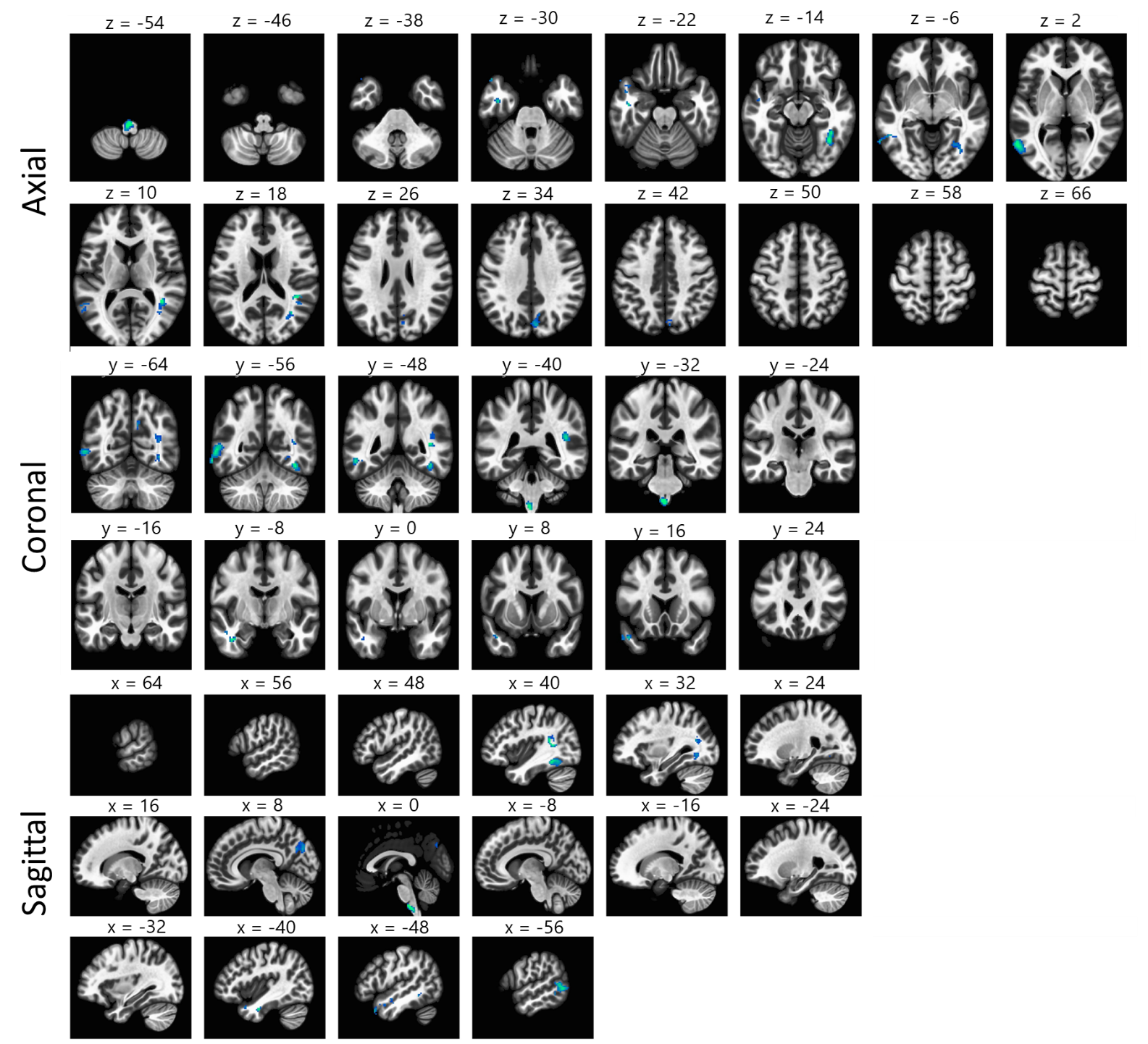
**

**Supplementary Figure 5.** Multivariate analysis of functional connectivity when viewing faces. Differences in whole-brain functional connectivity patterns between DPs and Controls using a section-based presentation of the significant cortical and subcortical areas.

**Supplementary Table 6.** Multivariate analysis of functional connectivity. Overlap of voxels with different pattern of functional connectivity when viewing flowers in the Control>DP contrast with regions from the cortical and subcortical Harvard Oxford atlas (<https://neuroconductor.org/help/ggsegHO/>).

| Region | Hemisphere | Overlapping voxels |
| --- | --- | --- |
| Precuneus cortex | both | 130 voxels |
| Lingual Gyrus | right | 97 voxels |
| Cerebellum Crus I | left | 83 voxels |
| Cerebellum lobule VI | left | 45 voxels |
| Cuneal Cortex | right | 34 voxels |
| Brainstem | both | 25 voxels |
| Brainstem | left | 22 voxels |
| Amygdala | right | 8 voxels |
| Temporal Occipital Fusiform Cortex | right | 5 voxels |
| Occipital Fusiform Gyrus | left | 5 voxels |
| Posterior Parahippocampal Gyrus | right | 3 voxels |
| Hippocampus | right | 2 voxels |
| Temporal Occipital Fusiform Cortex | left | 1 voxel |

**
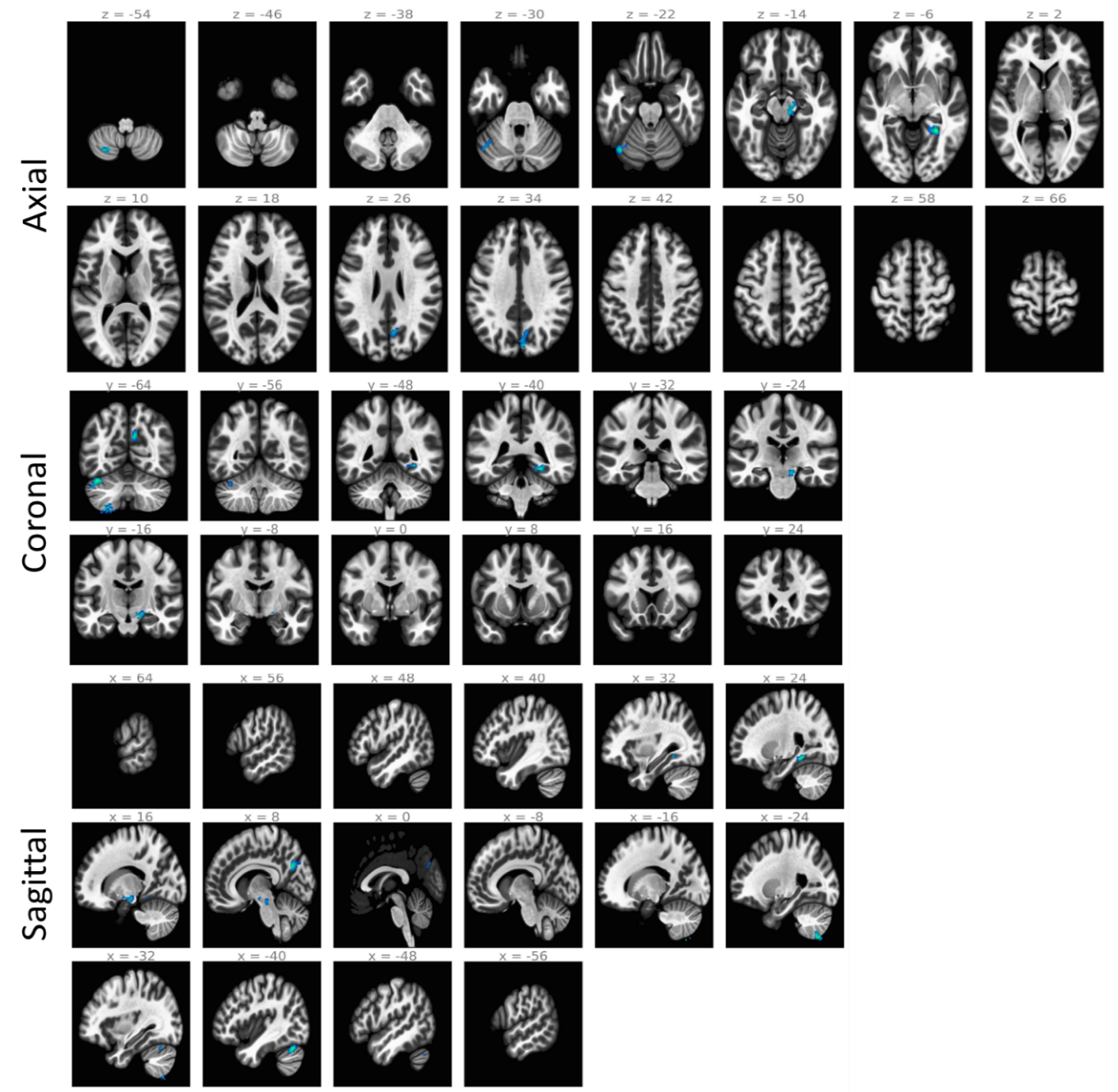
**

**Supplementary Figure 6.** Multivariate analysis of functional connectivity when viewing flowers. Differences in whole-brain functional connectivity patterns between DPs and Controls using a section-based presentation of the significant cortical and subcortical areas.

**
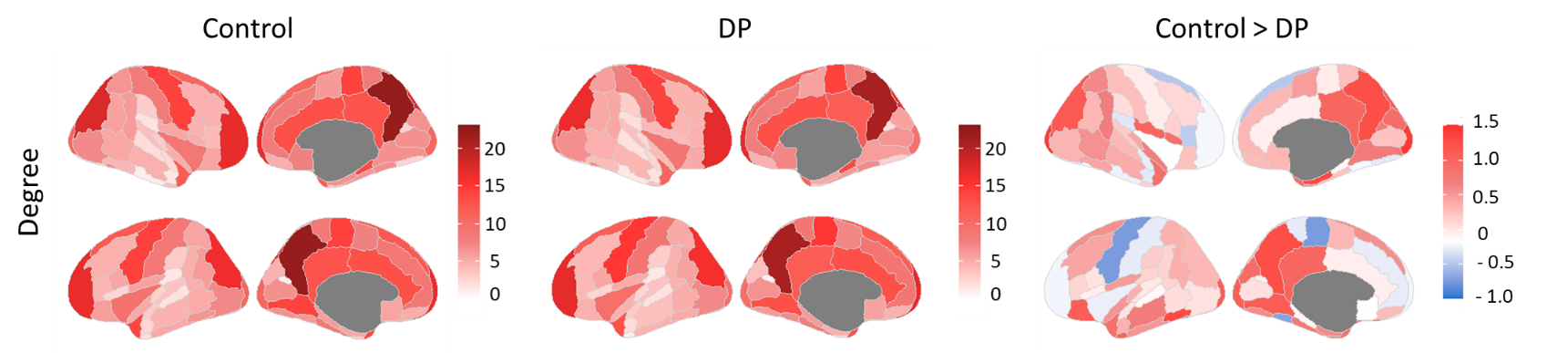


Supplementary Figure 7.** Region-level node degree in Control and DP groups, and Control > DP difference in node degree.
